# Supplementary figures and images for: Univariable and multivariable Mendelian randomization study identified the key role of gut microbiota in immunotherapeutic toxicity
Source: Eur J Med Res. 2024 Mar 12;29:161. doi: 10.1186/s40001-024-01741-7 (PMC10929167; doi:10.1186/s40001-024-01741-7)

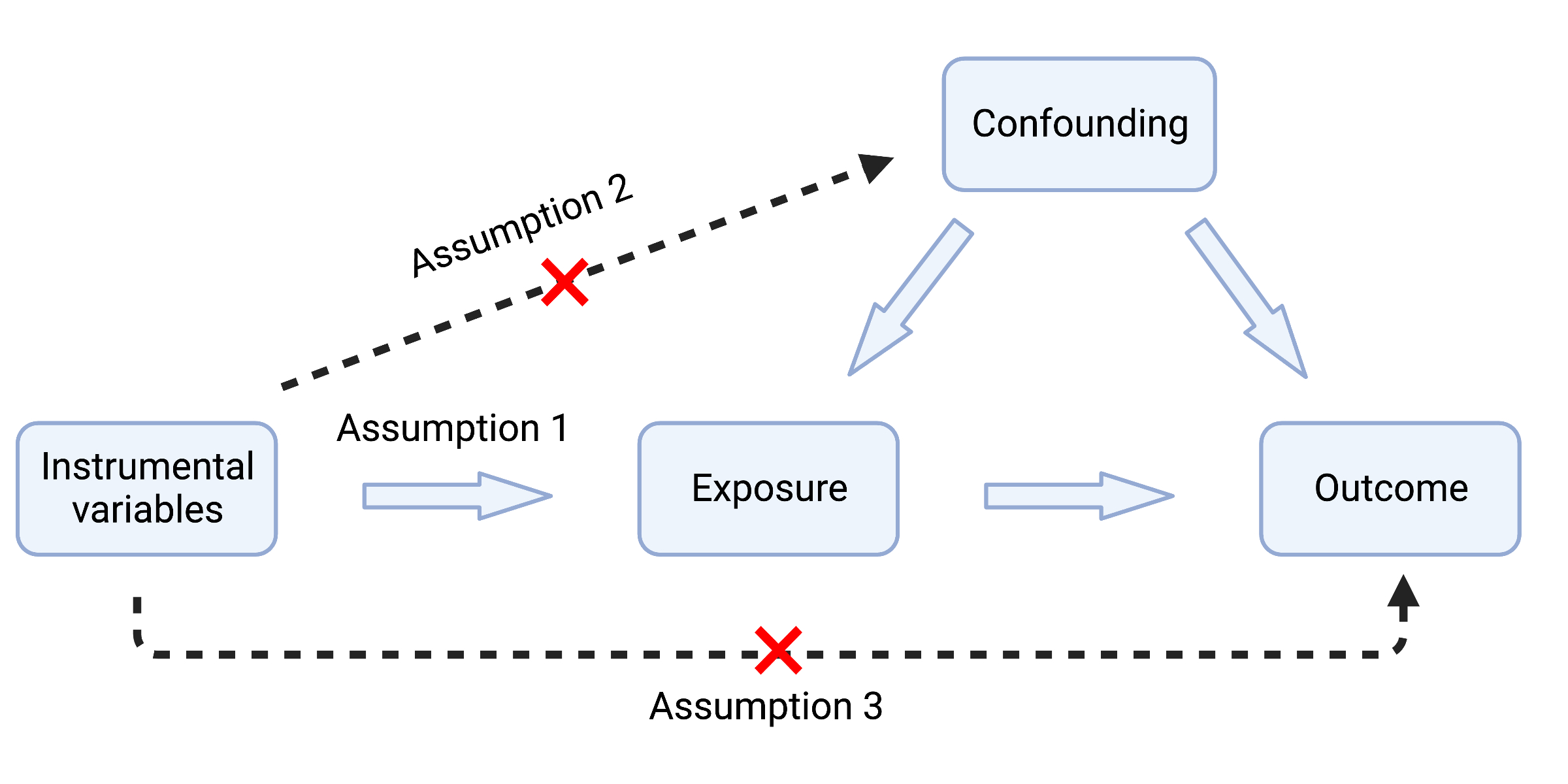

Supplement: Supplementary file 1 — Additional file 1: Figure S1. Illustration of MR assumptions [file 40001_2024_1741_MOESM1_ESM.jpg]
